# The infrastructural capacity of Ghanaian health facilities to provide safe abortion and post-abortion care: a cross-sectional study

**DOI:** 10.1186/s12913-021-07141-5

**Published:** 2021-10-15

**Authors:** Onikepe Owolabi, Taylor Riley, Easmon Otupiri, Chelsea B. Polis, Roderick Larsen-Reindorf

**Affiliations:** 1 Guttmacher Institute, 125 Maiden Lane, 7th floor, New York, NY 10038 USA; 2 Vital Strategies, 100 Broadway, 4th Floor, New York, 10005 USA; 3 Department of Population, Family and Reproductive Health, School of Public Health, Kwame Nkrumah University of Science and Technology, Kumasi, Ghana; 4 Department of Epidemiology, Johns Hopkins Bloomberg School of Public Health, 615 N Wolfe St, Baltimore, MD 21205 USA; 5 Department of Obstetrics & Gynaecology, School of Medicine and Dentistry, Kwame Nkrumah University of Science and Technology, Kumasi, Ghana

**Keywords:** Ghana, Abortion, Post-abortion care, Safe abortion care, Quality of care, Infrastructural quality

## Abstract

**Background:**

Ghana is one of few countries in sub-Saharan Africa with relatively liberal abortion laws, but little is known about the availability and quality of abortion services nationally. The aim of this study was to describe the availability and capacity of health facilities to deliver essential PAC and SAC services in Ghana.

**Methods:**

We utilized data from a nationally representative survey of Ghanaian health facilities capable of providing post-abortion care (PAC) and/or safe abortion care (SAC) (*n* = 539). We included 326 facilities that reported providing PAC (57%) or SAC (19%) in the preceding year. We utilized a signal functions approach to evaluate the infrastructural capacity of facilities to provide high quality basic and comprehensive care. We conducted descriptive analysis to estimate the proportion of primary and referral facilities with capacity to provide SAC and PAC and the proportion of SAC and PAC that took place in facilities with greater capacity, and fractional regression to explore factors associated with higher structural capacity for provision.

**Results:**

Less than 20% of PAC and/or SAC providing facilities met all signal function criteria for basic or comprehensive PAC or for comprehensive SAC. Higher PAC caseloads and staff trained in vacuum aspiration was associated with higher capacity to provide PAC in primary and referral facilities, and private/faith-based ownership and rural location was associated with higher capacity to provide PAC in referral facilities. Primary facilities with a rural location were associated with lower basic SAC capacity.

**Discussion:**

Overall very few public facilities have the infrastructural capacity to deliver all the signal functions for comprehensive abortion care in Ghana. There is potential to scale-up the delivery of safe abortion care by facilitating service provision all health facilities currently providing postabortion care.

**Conclusions:**

SAC provision is much lower than PAC provision overall, yet there are persistent gaps in capacity to deliver basic PAC at primary facilities. These results highlight a need for the Ghana Ministry of Health to improve the infrastructural capability of health facilities to provide comprehensive abortion care.

## Introduction

Ghana is one of few countries in sub-Saharan Africa with relatively liberal abortion laws [1]. Since 1985, abortion has been legal in cases of rape, defilement, incest, fetal abnormality or disease or to protect physical or mental health [1]. The Ghanaian government has attempted to expand access to safe abortion care (SAC) and post-abortion care (PAC) over the past 25 years via policies, clinical guidelines, and health workforce trainings [2–4]. However, access to high quality SAC is not universal in Ghana where there continues to be stigma around obtaining abortions, [5, 6] and poor knowledge of the abortion law by women [7, 8] and some providers [9]. Access to PAC remains essential in this context because complications can occur for women with miscarriages and induced abortions, [10] and a substantial number of Ghanaian women continue to experience unsafe induced abortions. A study using data from the 2017 Ghana Maternal Health Survey estimated that 64% of Ghanaian women who self-reported an abortion during the last five years described the conditions, procedures or providers of their abortion procedures as unsafe [11]. A recently published paper by Polis et al. (which used data from the same survey as the present analysis) estimated that 71% of induced abortions in Ghana were illegal [12]. While researchers have recommended decentralization of comprehensive abortion care (CAC)- which includes both PAC and SAC- to primary healthcare facilities as a strategy to increase access to CAC, [13–15] evidence from maternal health suggests that improving quality of care is more impactful than simply expanding access to care for women [16]. Providing access to high-quality SAC and PAC – which both require similar equipment and staffing - is essential to ensure women can meet their reproductive desires, while avoiding the morbidity and mortality associated with unsafe abortion.

Although some data exists on women’s experiences obtaining induced abortions in Ghana, an evidence gap remains around the availability and quality of SAC and PAC in Ghanaian health facilities. A study conducted by the Ghana Health Service and Ipas in 2007 found that 25% of public facilities reported providing PAC. Half of the public hospitals had two or more functional manual vacuum aspirators (MVAs), whilst 8% of primary care facilities have one or more functional MVAs [17]. Evidence from other low- and middle-income countries (LMICs) suggests that the capacity of primary level facilities to deliver basic PAC services is very low [18] and that PAC and SAC caseloads are much lower in primary-level facilities than in higher level facilities [14].

To date, studies on quality of reproductive healthcare in Ghana have focused on routine and emergency obstetric and newborn care, finding that, in the rural Brong Ahafo region, capacity to provide services was low [19–21]. While 68% of all births were in facilities, only 18% occurred in facilities rated as high quality [19–21]. Evidence from a maternal health study in five countries in sub-Saharan Africa suggests that lower delivery volume was consistently associated with poorer quality of care [22]. An important step towards improving quality of care is evaluating current quality of care and associated factors. However, there is minimal recent systematic evidence on the relationship between CAC quality and caseload volume in LMICs to guide policy decisions on how governments can balance the expansion of access to abortion care with ensuring consistent delivery of high-quality services within limited healthcare budgets [23, 24]. This decision may range from allowing all lower-level and lower patient volume health facilities to provide both components of CAC care (PAC and SAC) at one end (which expands geographic access considerably), to concentrating staffing and resources within fewer high-volume facilities to ensure high-quality care (which may include some, but not all primary-level facilities) at the other [24].

To inform policymakers aiming to increase access to high quality care, we conducted an analysis to describe the infrastructural capacity of health facilities to deliver essential PAC and SAC services in Ghana. Our goals were to estimate the proportion of primary and referral facilities with high infrastructural capacity in 2017, the proportion of SAC and PAC that took place in facilities with high infrastructural capacity, and the association between client volume, and infrastructural capacity to provide care accounting for other facility factors.

## Material and methods

### Sampling and inclusion

We utilized data from a nationally representative survey of Ghanaian health facilities capable of providing PAC and/or SAC [12]. The full details on sampling can be found in this paper [12].

In summary, from a list of all eligible health facilities reporting data through the District Health Management System in 2017 (*n* = 2758), we selected 608 facilities using a two-stage stratified cluster sampling design with four strata. One stratum, sampled with certainty, contained all teaching (*n* = 4) and regional (*n* = 10) hospitals. The other strata contained all remaining facility levels in each of Ghana’s three ecological zones. We selected facilities in the Northern zone at twice the rate of facilities elsewhere to enable computation of representative estimates in all zones, and calculated weights as nonresponse-adjusted inverses of known selection probabilities. We completed interviews in 539 (89%) of the sampled facilities.

In this analysis, we included only those 326 facilities that reported having at least one PAC patient (unweighted *n* = 317, 57% of sampled facilities) or SAC patient (unweighted *n* = 103, 19% of sampled facilities) in the prior 12 months. We only included facilities with patients in the last 12 months because we were trying to assess the actual capacity of facilities to provide PAC and SAC not their theoretical capability as data were collected on services provided within this period. Thus, if a facility had not delivered services, it would have null answers to all the relevant questions for analysis. Assessing theoretical capability would have involved asking questions about their ability to deliver services even if they have not provided them within the period of interest and is more likely to overestimate their overall functioning [25].

Our sampling frame did not include 21 NGO facilities that provide PAC or SAC. In the original study, we obtained caseloads from NGOs directly and did not conduct the full health facility assessment in these sites [12]. Using aggregated PAC and SAC caseloads provided by these NGOs, we found that most PAC cases were seen in non-NGO facilities (99%) and 25% of SAC cases were performed by NGOs [12].

### Data collection

At each facility, following informed consent, trained interviewers surveyed a senior personnel member knowledgeable about PAC and, where relevant, SAC at that facility. Interviews were conducted by 17 individuals (bachelors, masters or PhD students), each with several years’ experience conducting interviews on sexual and reproductive health issues, all of whom participated in an eight day training to ensure consistent data collection using standardized instruments were supervised by seven senior staff members [12]. All study activities were performed in accordance with the relevant guidelines and regulations at the facilities.

### Measures

#### Quality

In this study, we use infrastructural capability to provide PAC and SAC as a proxy measure for the quality of PAC and SAC within the health system. We adapted the signal functions approach for comprehensive abortion care published by Campbell et al. to evaluate infrastructural capability to deliver these services (Tables 1 and 2) [25]. Initially developed by the United Nations [26, 27] to evaluate the provision of emergency obstetric care, signal functions have been adapted for different healthcare interventions, including abortion [28]. The signal function approach typically consists of a shortlist of indicators to assess health facilities capability to provide the most effective or life-saving interventions for managing the most common complications within a health service area. Signal functions for abortion address the commonest signs and symptoms of serious abortion-related complications, which are severe hemorrhage and infection. These are typically due to incomplete abortion, infections related to incomplete abortion (e.g., septic abortions, severe systemic infection), or invasive procedures -- lacerations or perforations which may lead to peritonitis. To manage these complications (especially as they start to get more serious), the shortlist of indicators proposed for PAC by Campbell (which also overlap with emergency obstetric care signal functions proposed by the WHO) include -- removal of retained products of conception (using vacuum aspiration or medication abortion), ability to administer parenteral antibiotics (which is critical for women who are very ill and not table to ingest orally), fluid replacement, etc. (see Table 1).

**Table 1:** Criteria used to classify facilities as having full SAC or PAC capability

Capability to Provide Basic SAC *Functions expected of Primary-level Health Facilities*	Capability to Provide Comprehensive SAC *Functions expected of Referral-level Health Facilities*	Capability to Provide Basic PAC *Functions expected of Primary-level Health Facilities*	Capability to Provide Comprehensive PAC *Functions expected of Referral-level Health Facilities*
1. Performed vacuum aspiration for women obtaining SAC in the 3 months before the survey^1^ 2. Provided medication abortion to women obtaining SAC in the 3 months before the survey^2^ 3. Offers family planning during at least one day a week 4. Reported offering at least one modern, short-acting family planning method to post-abortion or abortion patients during the survey^4^ 5. 1 or more health professionals on duty at least once a week 6. For facilities without comprehensive PAC, reported capacity to communicate^5^ and/or refer to other facilities		1. Removed retained products of conception in PAC patients in the 3 months before survey^3^ 2. Administered parenteral antibiotics to PAC patients in the 3 months before survey 3. Administered parenteral uterotonics to PAC patients in the 3 months before survey 4. Administered intravenous fluids to PAC patients in the 3 months before survey 5. Offers family planning 7 days each week 6. Reported offering at least one modern, short-acting family planning method to post-abortion or abortion patients during the survey^4^ 7. Has staff trained in PAC on duty or on call 24 h, 7 days a week	
	7. Performed dilatation and evacuation for women obtaining SAC in the 3 months before the survey. 8. Reported offering at least one long-acting reversible method or permanent method to post-abortion or abortion patients during the survey^6^	8. Reported a functional and available means of communication for referring emergencies in the 3 months before the survey^5^ 9. Reported a functional and available ambulance or other vehicle with fuel (owned by facility of shared with other persons) that could be used to transport referred patients in the 3 months before the survey	10. Administered blood transfusions to PAC patients in the 3 months before survey 11. Reported having a functional operating room PAC in the 3 months before survey 12. Reported offering at least one long-acting reversible method or permanent method during the survey^6^

^*1*^*Vacuum aspiration included-manual vacuum aspiration and electric vacuum aspiration*

^*2*^*Medication abortion included- mifepristone and misoprostol combination or misoprostol alone*

^*3*^*Methods included in removal of retained products included- misoprostol and mifepristone, misoprostol alone, manual vacuum aspiration (MVA), electric vacuum aspiration (EVA)*

^*4*^*Modern short-acting methods included- fertility awareness based methods, male condom, female condom, pills, injectables, emergency contraception*

^*5*^*Means of communication included- landlines, mobile phones (including staff personal phones), and radios*

^*6*^*Long acting reversible methods or permanent methods included- Implants, intrauterine devices*

*Female sterilization, Male sterilization*

**Table 2:** Basic and comprehensive signal Function criteria for PAC and SAC, Ghana 2018

	Total		Facility type			
			Primary facilities		Referral facilities	
**Post Abortion Care**	Weighted N	%	Weighted N	%	Weighted N	%
***Basic and comprehensive***						
Remove retained products of conception	1044	73%	533	59%	512	96%
Administer parenteral antibiotics^1^	904	64%	452	51%	452	85%
Administer parenteral uterotonics	959	67%	504	56%	454	85%
Administer intravenous fluids	1060	74%	576	64%	483	91%
Offers family planning 7 days each week	1212	85%	790	88%	422	79%
Provide at least one modern, short-acting family planning method^2^	635	52%	459	57%	176	41%
Has staff trained in PAC on duty or on call 24 h, 7 days a week	879	61%	524	58%	355	67%
***Basic Only***						
Communicate with (other) referral facilities	1341	94%	821	91%	520	98%
Has vehicle with fuel to transport women needing referral elsewhere	983	69%	568	63%	415	78%
***Comprehensive only criteria (only in referral facilities)***						
Administer a blood transfusion^3^	–	–	–	–	326	61%
Reported having a functional operating room for PAC in the 3 months before survey	–	–	–	–	457	86%
Reported offering at least one long-acting reversible method or permanent method during the survey	–	–	–	–	402	75%
**Total Facilities providing PAC in the past year** ^**5**^	1432	57%	898	48%	533	87%
**Safe Abortion Care**						
***Basic and comprehensive***						
Perform vacuum aspiration	417	90%	208	83%	208	97%
Can provide medication abortion	422	91%	226	90%	196	91%
Offer family planning at least once a week	425	92%	228	91%	198	92%
Provide at least one modern short acting family planning method	418	90%	220	88%	198	92%
1+ health professionals on duty	465	100%	250	100%	215	100%
Communication or referral for facilities without comprehensive PAC	366	79%	199	79%	167	78%
***Comprehensive only criteria (only in referral facilities)***						
Can provide dilatation and evacuation for second trimester abortions	–	–	–	–	54	25%
Provide at least one long acting reversible or permanent method	–	–	–	–	189	88%
**Total Facilities providing SAC in the past year** ^**5**^	465	19%	250	13%	215	35%
**Facilities with a copy of Comprehensive Abortion Care guidelines on-site**^**4**^	379	24%	239	24%	140	26%
**Total number of sampled facilities providing PAC and/or SAC**	1550		1003		547	

Table presents nationally, representative weighted numbers and percentages

(1) 20 weighted missing responses on this variable

(2)205 weighted missing responses on this variable

(3)97 weighted missing responses on this variable

(4)Visually confirmed by interviewer. The denominator is facilities that reported providing PAC or SAC (last row)

(5)Out of all facilities in the sample

Overall, quality of care is a challenging concept to measure because it is a multi-dimensional construct. Studies evaluating quality of care have tended to utilize the Donabedian framework and attempted to evaluate one or more of the three theoretical domains of health service quality he described- structure or inputs to care, process or content of care, and the outcomes of care. Signal functions consist of key structural (input) indicators and past performance of interventions (process) indicators, and are typically defined for two levels of care within the health system: basic and comprehensive.

Basic care is defined as the minimum service that primary (and higher) facilities should be able to provide, and comprehensive care is defined as the minimum care referral facilities should be able to provide. Signal functions do not provide any information on the content or outcomes of the essential care indicators evaluated. As part of our adaptations, we did not include all the staffing signal functions that Campbell and colleagues recommend. For measurement of basic and comprehensive PAC, we assumed that our signal function of availability of staff trained in PAC on duty or on call 24 h, 7 days a week encompassed the following signal functions: the facility was open 24 h per day, 7 days per week with at least one health professional on duty for SAC and at least three health professionals registered for PAC. We also used the availability of a functional operating room as a proxy for surgical/laparotomy capability (as one of ten components fulfilling the definition for comprehensive PAC) because we did not collect data on whether the facility performed emergency laparoscopy, laparotomy, or hysterectomy in the three months preceding data collection. We assessed the capability of primary facilities to provide basic PAC and SAC and of referral facilities to provide basic and comprehensive PAC and SAC (Table 1). Abortion-related signal functions are a helpful approach of evaluating facility-level quality because they utilize a condensed list of indicators which can be easily collected as an additional module in surveys. Further, they are usually summarized into aggregate indicators of basic and comprehensive capability which are aligned with primary and higher-level care in each context. This makes it relatively easy to compare facility performance them across countries and with other maternal health services that have been similarly assessed.

We sought guidance for study planning and dissemination from our Technical Advisory Committee, which included community representatives and technical experts. We also evaluated the proportion of facilities that had copies of the most recent Ghana Health Service comprehensive abortion care (CAC) guidelines [3] available on site as these guidelines lay out how high quality abortion care is intended to be provided within the Ghana Health Service.

#### Facility level

We classified health centers, health clinics, and maternity homes as primary facilities and we classified teaching, regional, district/university, and other hospitals as referral facilities [29].

#### PAC and SAC caseloads

We elicited information on the number of patients who received PAC or SAC in each facility in the last year; details on how we calculated caseloads are published elsewhere [12].

Drawing on the approach used by Kruk et al., [22] in an analysis with similar objectives, we grouped PAC caseloads into the following categories: ≤ one PAC patient per month (i.e. ≤12 per year); up to one per week (i.e. 13–52 per year); up to two per week (i.e. 53–104 per year); ≥ three per week (i.e. ≥105 per year). For SAC caseloads, we used similar categories but combined the first and second of these categories, as few SAC-providing facilities reported ≤ one SAC patient per month.

### Statistical analysis

We determined frequencies of each signal function criterion met for primary (basic only) and referral facilities (basic and comprehensive). For each signal function, we assigned a 1 if the function was available and a 0 if not. If data was missing the facility received a score of zero. We computed total scores indicating each facility’s availability of every signal function item out of the total theoretical availability for each capacity level: basic PAC (maximum score 9) or SAC (maximum score 6) and comprehensive PAC (maximum score 10) or SAC (maximum score 8).

For each category of PAC and SAC, we converted each facility’s total score into a proportion out of 1 by dividing by the possible maximum score for each category to generate a proportional capacity score. After calculating percentiles of these proportions, we created categories of infrastructural capacity using the following thresholds: low capacity (<50th percentile), medium capacity (50th–79th percentile), or high capacity (≥80th percentile).

To examine factors associated with the capacity to provide care, we conducted fractional outcome regressions using the continuous proportional capacity scores for basic and comprehensive PAC and SAC as the dependent variables. We investigated associations between capacity scores and all the following facility characteristics: PAC and SAC caseloads, managing authority (public or private/faith-based), location (urban/rural), and presence of one or more staff trained in manual vacuum aspiration (MVA) or electric vacuum aspiration (EVA) in one model. Descriptive statistics and regression models were weighted for the complex survey design. Estimation of confidence intervals incorporated sample stratification and clustering. We conducted analyses in Stata 16.0 [30].

## Results

Although Ghana has made substantial efforts to improve access to PAC and SAC, over half of non-NGO facilities reported providing care for PAC (57%) while fewer reported providing SAC (19%) patients in the preceding 12 months. Among PAC-providing facilities, referral facilities had greater capacity to provide most signal functions than primary facilities (Table 2). For instance, 96% of referral facilities had capacity to remove retained products of conception versus 59% of primary facilities. An exception was for family planning related functions: a higher proportion of primary facilities offered family planning seven days a week (88%) and provided one modern, short-acting method (57%) compared to referral facilities (79 and 41%, respectively). Overall, less than 60% of primary facilities reported the capability to provide signal functions related to the specific treatment services within basic PAC (results not shown). For comprehensive PAC signal functions, a higher proportion of facilities were able to provide the requisite services to treat complications. In addition to the services under basic PAC, around two-thirds (61%) of referral facilities could provide blood transfusions, 75% reported offering one long-acting reversible or permanent contraceptive method, and 86% reported having a functional operation room. The ability to meet staffing requirements for PAC appeared challenging for many facilities and only 57% of primary facilities and 41% of referral facilities had PAC trained staff on call 24/7.

In contrast, capacity to provide basic SAC signal functions was similar between primary and referral facilities, and staffing requirements for SAC were more easily met than for PAC. However, only one-quarter of SAC-providing referral facilities could perform dilatation and evacuation for second trimester abortions. Out of all facilities in the sample that reported offering PAC and/or SAC, only 24% had the CAC guidelines on-site.

**Fig. 1:**
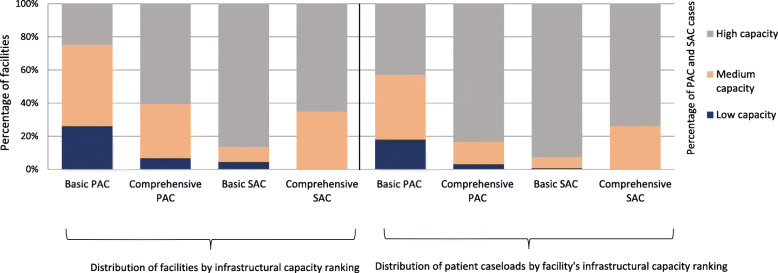
Distribution of facilities and caseloads by capacity ranking, for basic and comprehensive PAC and SAC. Notes: Basic level SAC and PAC capacity are out of primary facilities, and comprehensive PAC and SAC capacity is out of referral facilities

Less than 20% of PAC and/or SAC providing facilities were able to provide all the signal functions for basic or comprehensive PAC and comprehensive SAC (results not shown). Half of PAC-providing primary facilities were classified as medium capacity for PAC (49%), whilst most SAC-providing primary facilities (86%) and PAC- (61%) and SAC-providing (65%) referral facilities were classified as high capacity (Fig. 1). A disproportionate number of cases, particularly for PAC, were treated at facilities classified as high capacity (Fig. 1), but still, less than half (43%) of PAC cases treated in primary facilities were treated in a facility classified as high capacity, whereas in referral facilities, the majority of PAC cases were treated in high-capacity facilities (83%). Conversely, almost all SAC cases at primary facilities were treated in facilities classified as high capacity (93%) while the proportion of SAC cases treated in referral facilities classified as high capacity was 74%.

In multivariable analysis, higher PAC caseloads were significantly associated with higher basic and comprehensive capacity in primary and referral facilities, respectively (Table 3). For example, compared with the reference group of one PAC patient per month or less, having up to one PAC patient per week increases the capacity for care score by 0.07 (95% CI: 0.02, 0.12) while greater than two PAC patients per week increases the capacity score by 0.28 (95% CI: 0.15, 0.42). Having a staff member trained in MVA/EVA was associated with higher basic PAC capacity in primary facilities by 0.12 (95% CI: 0.07, 0.18) (Table 3). In referral facilities, private or faith-based ownership was associated with lower comprehensive capacity by 0.07 (95% CI: − 0.13, 0.01) compared to government facilities. Referral facilities located in rural areas were associated with higher comprehensive capacity. Having a staff member trained in MVA/EVA was also associated with higher comprehensive PAC capacity by 0.23 (95% CI: 0.11, 0.35). While SAC caseloads, managing authority, and annual number of deliveries were not significantly associated with basic or comprehensive SAC capacity in primary or referral facilities, being in a rural area was associated with lower basic SAC capacity.

**Table 3:** Fractional regression models showing facility characteristics associated with PAC and SAC proportional capacity scores

PAC					SAC				
	Basic PAC		Comprehensive PAC			Basic SAC		Comprehensive SAC	
	Primary facilities		Referral facilities			Primary facilities		Referral facilities	
Covariates	Marginal effects† (95% CI)		Marginal effects† (95% CI)		Covariates	Marginal effects† (95% CI)		Marginal effects† (95% CI)	
**PAC Caseloads**					**SAC Caseloads**				
One PAC patient per month or less	1.00 (ref)		1.00 (ref)						
Up to one PAC patient per week	0.07 (0.02–0.12)**		0.13 (0.05–0.21)**		Up to one SAC patient per week	1.00 (ref)		1.00 (ref)	
Up to two PAC patients per week	0.08(−0.04–0.21)		0.13 (0.05–0.22)**		Up to two SAC patients per week	0.05(−0.01–0.11)		0.00(− 0.08–0.09)	
Greater than two PAC patients per week	0.28 (0.15–0.42)***		0.14 (0.04–0.24)**		Greater than two SAC patients per week	0.03(− 0.08–0.14)		− 0.02(− 0.13–0.09)	
**Managing authority**					**Managing authority**				
Public	1.00 (ref)		1.00 (ref)		Public	1.00 (ref)		1.00 (ref)	
Private or faith-based	0.03(−0.04–0.10)		−0.07(− 0.13 – − 0.01)*		Private or faith-based	−0.05(− 0.12–0.02)		−0.08(− 0.18–0.02)	
**Residence**					**Residence**				
Urban	1.00 (ref)		1.00 (ref)		Urban	1.00 (ref)		1.00 (ref)	
Rural	0.00(−0.06–0.06)		0.07 (0.01–0.13)*		Rural	− 0.08(− 0.14 – − 0.01)*		0.03(− 0.04–0.10)	
**Number of deliveries per year**	0.00(−0.00–0.00)		0.00 (0.00–0.00)*		**Number of deliveries per year**	0.00(− 0.00–0.00)		0.00(−0.00–0.00)	
**Staff training in vacuum aspiration**‡									
No staff trained	1.00 (ref)		1.00 (ref)						
One or more staff trained	0.12 (0.07–0.18)***		0.23 (0.11–0.35)***						

† Marginal effects of covariates represent changes in the proportional quality score for basic PAC and SAC and comprehensive PAC and SAC compared with the reference group for each covariate

‡ Either manual (MVA) or electric (EVA)

*p < .05, **p < .01, ***p < .001

## Discussion

To our knowledge, this is the first nationally representative study examining the system-level infrastructural capacity for PAC and SAC provision in Ghanaian health facilities using a signal functions approach. Less than 20% of PAC and/or SAC providing facilities could provide all signal functions for basic or comprehensive PAC or for comprehensive SAC. Our results are similar to evidence from Zambia, which like Ghana has a relatively liberal abortion law, [25] but a recent study reports very low capability of facilities to provide basic and comprehensive PAC and SAC using similar signal functions criteria, and lower provision of SAC compared with PAC despite the law [31].

Although referral facilities had greater capacity than primary facilities to provide most of the basic SAC and PAC functions, primary facilities reported greater capability pertaining to family planning offerings. This is similar to findings in other low- and middle-income countries [18]. It is important that a range of contraceptive methods is available and services are provided at the same location where PAC or SAC takes place, to ensure sufficient method choice where women are counselled so that they can choose a method voluntarily, and to protect against future unintended pregnancies [32].

The association between basic and comprehensive PAC capacity and caseload is similar to findings from other African countries examining delivery volume and obstetric care quality [22]. Women may be more likely to go to facilities they perceive as providing higher quality care, [33–35] and this reputation may be connected to the availability of key infrastructure and staff [36]. Indeed, we observed strong associations between the presence of staff trained in MVA/EVA and greater PAC capacity. It is also possible that with higher PAC caseloads, staff can practice PAC provision skills, and their facilities may be more likely to stock required commodities and employ sufficient staff. That said, there is no systematic evidence on the relationship between PAC caseload and higher capacity to provide care elsewhere in sub-Saharan Africa. In referral facilities, PAC capacity was associated with being public and rural location; potentially because the most common referral hospitals in our sample are district hospitals. These are generally public facilities and are located in rural districts even though their exact geographical locations may be more urbanized than their catchment area.

Our study had several limitations. First, we did not include NGO facilities, which managed 25% of the SAC caseload in Ghana, [12] so we are missing an important contributor of SAC provision in our description of SAC quality. Second, we did not have sufficient data on staffing and training to examine the capability to deliver emergency surgical care in referral facilities as part of comprehensive PAC and we omitted asking questions to assess if staff had received SAC training. Third, we also did not ask to see commodities, verify if equipment was available and functioning, or confirm process-of-care indicators (e.g. ability to remove retained products of conception) by provider observation, which would have been more objective than verbal reports by respondents. Thus, this analysis may overestimate the capability of facilities to provide PAC and SAC. Fourth, although we examined the relationship between capacity and caseloads, we did not have data on adverse outcomes to examine the relationship between structural capacity and outcomes in women obtaining PAC and SAC. Fifth, because the signal function approach focuses on the availability of inputs and commodities, it incorporates limited measures of process, and does not examine patients’ perspectives on the care they received or the occurrence of adverse clinical outcomes. Recent evidence available on quality of care measurement from maternal health across the theoretical domains of structure, process and outcomes suggests that availability of essential infrastructure correlates poorly with provision of evidence-based care to patients [37]. Additionally, in its current form, signal functions for PAC and SAC do not include the third (and often neglected) component of the original PAC model- linkage to comprehensive reproductive health care services [38]. There is an opportunity for health systems to provide more holistic care by encouraging providers to provide women with information about other reproductive care services available at health facilities and referring them for those, which they might benefit from after obtaining PAC or SAC treatment. Despite the limitations of the signal functions approach, there are no published validated measures of the quality of abortion care incorporating all the conceptual domains outlined by Donabedian and other researchers in low- and middle-income contexts [39]. As researchers continue to develop and test standardized indicators to measure all domains of quality, this approach offers a relatively simple and familiar approach to examine structural and process quality in contexts where institutional capacity to deliver abortion services is not always assured, and evidence of gaps can facilitate advocacy for evidence-based policies to improve health outcomes [39].

## Conclusion

In summary, while fewer non-NGO facilities report providing SAC than PAC, the inputs to provide basic SAC are not universally available at primary or referral facilities reporting SAC provision. In addition, amongst facilities that provide PAC, there are gaps in capacity to deliver basic and comprehensive PAC. These results highlight a need for the Ghana Ministry of Health to improve the infrastructural capability of health facilities to provide CAC services. We recommend that the Ministry of Health ensures that the national CAC guidelines are available and implemented in all facilities. Increasing access to SAC is logistically achievable for Ghana, as SAC requires similar equipment and staffing as PAC, which more health facilities reported providing. Also, improving PAC quality is necessary because it is an essential emergency service that can mitigate the adverse effects of complications from miscarriages and unsafe abortions, which are still prevalent in Ghana [12]. Additionally, routine tracking and monitoring systems which incorporate relevant indicators in district health information and hospital supply chain systems should be organized to consistently evaluate the infrastructural capability of facilities to deliver comprehensive abortion care. More evidence on the relationship between caseload and greater quality is needed to guide policy decisions about the location and distribution of PAC and SAC provision. Future studies on quality of abortion care in Ghana should also incorporate data on adherence to evidence-based recommendations and patient outcomes, including their experiences of care. More holistic measures of quality are essential to improve the performance of the health system and achieve better reproductive health outcomes amongst women in Ghana and other low- and middle-income countries.

## Data Availability

The datasets generated and/or analyzed during the current study are not publicly available as they contain sensitive information about health facilities. De-identified versions of the raw Health Facilities Survey and Knowledgeable Informants Survey datasets collected by the authors and used in this analysis are available from Guttmacher Institute on reasonable request to researchers who wish to use the data for scholarly analysis. To discuss obtaining copies of these datasets, please contact popcenter@guttmacher.org with the detailed protocol for your proposed study, and information about the funding and resources you have to carry out the study.

## Abbreviations

CAC: Comprehensive abortion care
EVA: Electric Vacuum Aspiration
IRB: Institutional Review Board
LMICs: Low- and middle-income countries
MVA: Manual Vacuum Aspiration
NGO: Non-governmental organization
PAC: Postabortion Care
SAC: Safe abortion care
